# A near-infrared spectroscopy dataset of coal and coal-measure rock under diverse conditions

**DOI:** 10.1038/s41597-024-03422-w

**Published:** 2024-06-14

**Authors:** Yuanbo Lv, Shibo Wang, En Yang, Shirong Ge

**Affiliations:** 1 School of Mechanical and Electrical Engineering, China University of Mining and Technology, Xuzhou, 221116 China; 2 Jiangsu Province and Education Ministry Co-sponsored Collaborative Innovation Center of Intelligent Mining Equipment, China University of Mining and Technology, Xuzhou, 221116 China; 3 State Key Laboratory of Intelligent Mining Equipment Technology, China University of Mining and Technology, Xuzhou, 221116 China; 4 School of Intelligent Manufacturing, Jiangsu Vocational Institute of Architectural Technology, Xuzhou, 221116 China; 5 China University of Mining and Technology-Beijing, Beijing, 100083 China

**Keywords:** Energy access, Coal

## Abstract

The identification technology for coal and coal-measure rock is required across multiple stages of coal exploration, mining, separation, and tailings management. However, the construction of identification models necessitates substantial data support. To this end, we have established a near-infrared spectral dataset for coal and coal-measure rock, which includes the reflectance spectra of 24 different types of coal and coal-measure rock. For each type of sample, 11 sub-samples of different granularities were created, and reflectance spectra were collected from sub-samples at five different detection azimuths, 18 different detection zeniths, and under eight different light source zenith conditions. The quality and usability of the dataset were verified using quantitative regression and classification machine learning algorithms. Primarily, this dataset is used to train artificial intelligence-based models for identifying coal and coal-measure rock. Still, it can also be utilized for regression studies using the industrial analysis results contained within the dataset.

## Background & Summary

The stable and safe supply of coal is crucial for sustaining production and daily life^1^. It is necessary to constantly enhance the technical level of coal production, in which coal and coal-measure rock identification play a pivotal role throughout the entire coal production process. During the exploration stage^2^, identification technology aids engineers in rapidly determining the optimal locations, depths, and angles for drilling. During the mining stage^3^, it enables the shearer to follow the roof, which reduces equipment wear. During the separation stage^4^, it effectively distinguishes between coal and coal-measure rock, which in turn reduces energy consumption during subsequent processing stages. During the tailing management stage^5^, unutilized coal can be effectively identified and recovered, thereby minimizing waste. Currently, there are various methods for coal and coal-measure rock identification, such as imaging^6^, gamma-ray^7^, and radar^8^ techniques. In recent years, spectral-based methods have gained widespread attention due to their advantages in speed and efficiency^9^. Spectroscopy reveals molecular composition and structure through absorption spectra of vibrations at fundamental and overtone frequencies. Spectroscopy serves as a “fingerprint” for different substances^10^.

Traditionally, identification models relied on database matching methods^11^. However, with the advancement of artificial intelligence (AI) technology, AI-based identification models have achieved superior performance. Currently, various classification models have been developed using advanced machine learning techniques, including Convolutional Neural Network (CNN)^12,13^, Broad Learning System (BLS)^14^, and Bidirectional Long Short-Term Memory network (Bi-LSTM)^15^, as well as pre-trained Vision Transformer (ViT)^16^. Notably, Gaussian Support Vector Machine (SVM)^17^, Linear Discriminant Analysis (LDA)^18^, BLS^19^, Random Forest (RF)^20^, and Extreme Learning Machine (ELM)^21^ have also been utilized to establish models for classifying coal types and provenance. Specifically, for the quantitative analysis of coal composition, researchers have employed methods such as XGBoost^22^ and Partial Least Squares (PLS)^23^ to construct regression models.

While AI-based models are widely used, their practical implementation introduces new challenges, notably the need for diverse training data. Numerous spectral databases have been established, such as the USGS Spectral Library Version 7 by the United States Geological Survey^24^, primarily featuring mineral reflectance spectra covering visible, near-infrared, and mid-far infrared bands of absorption and reflectance spectra; NASA JPL’s ECOSTRESS Spectral Library - Version 1.0^25^, which focused on minerals and rocks across the 0.4~15.4 μm wavelength range; The “ 2D hyperspectral library of mineral reflectance” by Laurent Fasnacht^26–29^ includes reflectance spectra of minerals in near-infrared ranges. These databases provide a valuable data platform for researchers, but they are primarily intended to support fields like geological exploration, environmental monitoring, agriculture, and forestry.

**Fig. 1:**
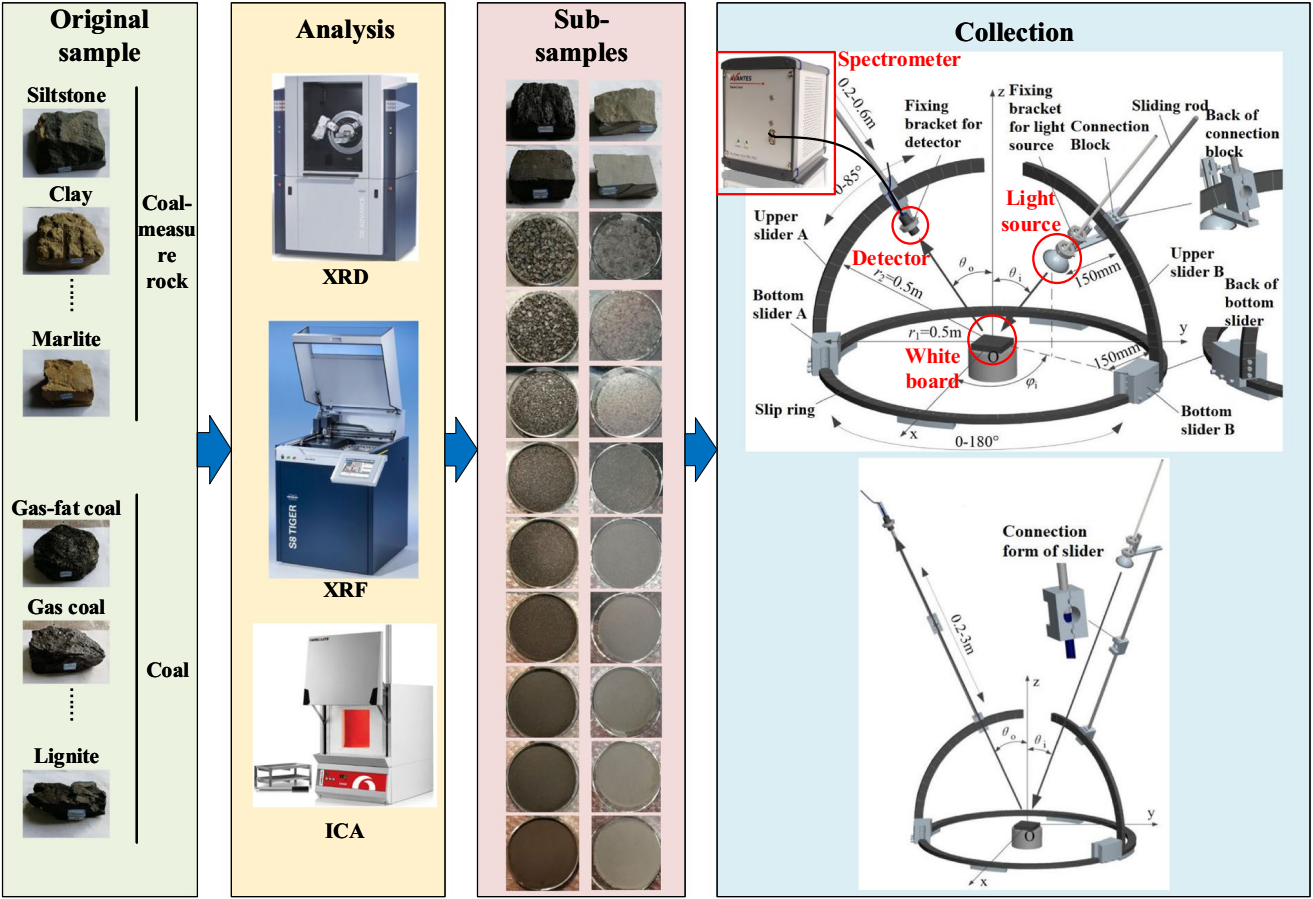
Overview of coal and coal-measure rock sample preparation experiment and spectral acquisition.

The primary subjects of identification research are coal and coal-measure rock, where coal-measure rock refers to sedimentary rocks like mud shale, sandstone, and limestone. Reflectance spectra can differ due to geological variations and types in different regions^30^. Even within the same coal mine, reflectance spectra of the same type of coal and coal-measure rock can vary significantly due to differences in detection geometry, such as angle and distance. As well as coal and coal-measure rock characteristics like granularity and surface roughness^31^. Therefore, a specialized spectral dataset for coal and coal-measure rock identification is needed. We have collected coal and coal-measure rock samples from various mining areas in China, including 12 types of coal-measure rocks and 12 types of coal. Each coal sample underwent XRF (X-ray Fluorescence), XRD (X-ray Diffraction), and ICA (Industrial Component Analysis) treatments. Each collected sample was prepared into 11 different granularity sub-samples, and the reflectance spectra for all sub-samples were obtained using five different detection azimuth *ϕ*_*i*_, 18 different detection zenith *θ*_*i*_, and nine different light source zenith *θ*_*o*_. Fig. 1 show the overview of coal and coal-measure rock sample preparation experiment and spectral acquisition.

## Methods

### Coal and coal-measure rock samples collection

**Fig. 2:**
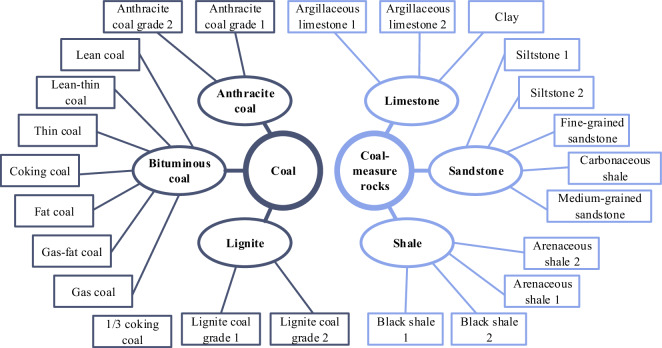
All the types of the coal and coal-measure rock.

We collected coal and coal-measure rock samples from different mining areas in various provinces of China using two collection methods: direct acquisition from fully mechanized mining faces and roof drilling. In total, we obtained representative samples of 12 types of coal, including anthracite coal, bituminous coal, and lignite, as well as 12 types of coal-measure rocks, including shale, sandstone, and limestone, as show in Fig. 2. To preserve the original surface morphology of the coal and coal-measure rock, the collected samples were promptly placed in self-sealing plastic bags and sealed for storage.

### Component analysis and sub-sample preparation

Component analysis was performed on each sample to investigate the material composition, which produces the spectral characteristics of coal and coal-measure rock reflectance. Three methods were employed for the study: XRD, XRF, and ICA. XRD determined the carbonaceous material structure and mineral composition type of the coal, XRF quantitatively measured the elemental content in the coal, ICA analyses determined the air-dried moisture (Mad), ash content (Aad), volatile matter (Vad), and fixed carbon (FCad) of the coal samples. Table 1 provides information on the instruments used in these three methods. Table 2 presents the configuration for the component analysis of each sample.

**Table 1:** Instrument information used for XRD, XRF, and ICA.

Analysis method	Instrument	Producer
XRD	Bruker D8 Advance X-ray Diffractometer	Germany
XRF	Bruker S8 Tiger X-ray Fluorescence Spectrometer	Germany
ICA	FD115 drying oven from Binder	Germany
	BS124S electronic balance from Sartorius	Germany
	AAF12/18 muffle furnace	UK
	VMF10/6 muffle furnace	UK

**Table 2:** Configuration of composition analysis for each sample.

Type(coal)	XRD	XRF	ICA	Type(coal-measure rock)	XRD	XRF	ICA
Anthracite coal grade 2	●	●	●	Argillaceous limestone 1	●	●	○
Anthracite coal grade 1	●	●	●	Argillaceous limestone 2	●	●	○
Lean coal	●	●	●	Siltstone 1	●	●	○
Lean-thin coal	●	●	●	Siltstone 2	●	●	○
Thin coal	●	●	●	Clay	●	●	○
Coking coal	●	●	●	Carbonaceous shale	●	●	○
Fat coal	●	●	●	Fine-grained sandstone	●	●	○
1/3 coking coal	●	●	●	Black shale 2	●	●	○
Gas-fat coal	●	●	●	Black shale 1	●	●	○
Gas coal	●	●	●	Medium-grained sandstone	●	●	○
Lignite coal grade 1	●	●	●	Arenaceous shale 1	●	●	○
Lignite coal grade 2	●	●	●	Arenaceous shale 2	●	●	○

Since ICA primarily involves measuring the chemical composition parameters of samples through combustion. The coal industry’s core focus is on coal, rock is considered additional materials produced during mining and are discarded after extraction. The purpose of conducting ICA is to sort coal samples based on chemical composition parameters, in order to classify them by different combustion levels, making this analysis exclusive for coal.

Due to the rough surface condition and particle size of coal and coal-measure rock, which are important factors affecting the spectral reflectance characteristics. For each type of coal and coal-measure rock, block samples with two different surface roughness levels and powdered samples with nine different particle sizes were prepared. The block samples were prepared, with one side having an *in-situ* fractured rough surface, the other having a relatively smooth surface polished to a surface roughness (Ry) less than 0.3 mm. The powdered samples of each coal and coal-measure rock type were obtained by standard sieving, resulting in nine different particle sizes: 8000 μm, 4750 μm, 2500 μm, 1000 μm, 500 μm, 210 μm, 100 μm, 74 μm, and 45 μm.

### Spectral data collection

Based on the relative positional relationship of samples, detectors, and light sources in spherical coordinates, a spherical coordinate-based reflectance spectroscopy measurement platform was designed. The three-dimensional model of the platform is shown in Fig. 1, which mainly includes the platform’s structure, whiteboard, detector, spectrometer, and light source; their parameters are shown in Table 3.

**Table 3:** Information of reflectance spectroscopy measurement platform.

Module	Attribute	Value	Module	Attribute	Value
Structure of platform	Radius	0.5 m	Detector	Type	Optical fiber collimator mirror
	Detection azimuth	0–360°		Field of view	0.12°
	Light source zenith	0–85°		Diameter	2.5 cm
	Detection zenith	−85°−85°	Spectrometer	Company	Avantes
	Distance of light source	0.2–0.6 m		Wavelength	1000–2500 nm
	Distance of detector	0.2–0.6 m		Spectral resolution	8.9 nm
Whiteboard	Material	PTFE	Light source	Type	Halogen lamp

The specific collection process is as follows:

1. Connect one end of the optical fiber to the detector and the other to the spectrometer.
2. Connect the spectrometer to the computer via USB and launch the AvaSoft software developed by Avantes.
3. Measure the radiance illuminance of complete reflection from a fixed position (two distances are 0.5 m, light source zenith of 45°, detection zenith of 0°, and detection azimuth of 0°).
4. Measure the zero radiance illuminance with the detector covered.
5. Collect the irradiance of the sample and calculate the reflectance spectrum using Formula 1.

1 $$R\left(\begin{array}{ccc}{\theta }_{i} & {\theta }_{o} & {\varphi }_{i}\end{array}\right)=\frac{S\left(\begin{array}{ccc}{\theta }_{i} & {\theta }_{o} & {\varphi }_{i}\end{array}\right)-{S}_{dark}\left(\begin{array}{ccc}\frac{\pi }{4} & 0 & 0\end{array}\right)}{{S}_{ref}\left(\begin{array}{ccc}\frac{\pi }{4} & 0 & 0\end{array}\right)-{S}_{dark}\left(\begin{array}{ccc}\frac{\pi }{4} & 0 & 0\end{array}\right)}$$

Where R represents the reflectance, S represents the radiance illuminance. The subscripts “ref” and “dark” indicate the radiance illuminance under complete reflection and detector covered, respectively. The parameters *θi, θ*_*o*_, and *φi* represent detection zenith, light source zenith, and detection azimuth.

During the spectrum collection process, the distance between the light source and the sample, as well as between the detector and the sample, is set to 0.5 meters. Five different detection azimuth (0°, 10°, 20°, 30°, 40°), eighteen different light source zenith (0°, 5°, 10°, 15°, 20°, 25°, 30°, 35°, 40°, 45°, 50°, 55°, 60°, 65°, 70°, 75°, 80°, 85°), and nine different detection zenith (10°, 20°, 30°, 40°, 45°, 50°, 60°, 70°, 80°) were set. These three angles were combined in a factorial design, resulting in 32 detection geometries. Thus, a total of 53 reflectance spectra were obtained for each type of sample in each condition. In total, 24 types of samples yielded 10496 reflectance spectra.

## Data Records

**Fig. 3:**
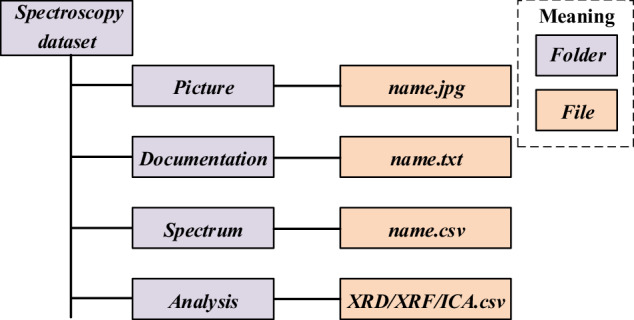
Structure of the data included in spectroscopy dataset.

The near-infrared spectroscopy dataset has been deposited into the Zenodo(10.5281/zenodo.11137126)^32^. The directory structure is shown in Fig. 3. The dataset includes a total of 4 folders: Photos, Documentation, Spectra, and Analysis. In the Spectra folder, there are exterior images of all samples in.jpg format. In the Documentation folder, there are text files (.txt) summarizing the origin of each sample, as well as ICA, and XRF results. The Spectra folder contains the reflectance spectrum of each sub-sample at diverse conditions in.csv format. Lastly, in the Analysis folder, there are.csv files containing the results obtained from XRF, XRD, and ICA for each sample. The names of the files in the first three folders correspond to their respective samples.

## Technical Validation

To ensure the reliability of the reflectance spectrum collected under diverse conditions, we conducted detailed inspections before, during, and after collection. The specific inspection methods are as follows:

1. Before collection, three individuals who deeply understood coal and coal-measure rock spectra were assigned different tasks. They were responsible for sample selection according to the labels, adjusting the measurement platform’s collection parameters, and operating the computer software. This division of labor helped to minimize the probability of errors.
2. During collection, for each sub-sample and each condition, the spectrometer was set to automatically acquire the reflectance spectrum ten times and then take the average. This approach can avoid distortions caused by a single spectral collection.
3. After collection, interpolation was applied to the exported reflectance spectrum using Avasoft. This step aims to obtain a reflectance spectrum with a wavelength interval of 1 nm and fill in any missing wavelength. By following these meticulous procedures, we ensured the accuracy and reliability of the collected reflectance spectrum for our dataset.

To validate the feasibility of the constructed dataset. In this manuscript, the analysis of reflectance spectra will be conducted using three approaches: binary classification of coal and coal-measure rock, multi-class classification of coal and quantitative regression of coal’s composition. This manuscript will validate the existing algorithms mentioned in the background & summary section. CNN, BLS, and Bi-LSTM will be utilized for the binary classification of coal and coal-measure rock using all spectral data. For the multi-class classification of coal, SVM, LDA, BLS, RF, and ELM will be employed using all spectral data of coal. Quantitative regression of coal’s composition will be performed using XGBoost and PLS using all spectral data of coal. During the dataset validation process, the dataset is divided into training set, testing set, and validation set in a 7:2:1 ratio. The algorithms were trained on a machine with an i7-8750H CPU, Quadro P1000, and Windows 10. The hyperparameters for all algorithms were initially set to their recommended appropriate values, and the specific training parameters can be found in the publicly available GitHub repository listed in the code availability section. The versions of the libraries used for each algorithm are detailed in Table 4.

**Table 4:** The library and version used by the algorithm environment.

Library	Version	Library	Version
python	3.8	torch	2.0.1
sklearn	1.1.2	numpy	1.24.4
xgboost	2.0.3	hpelm	1.0.10

To further validate the stability of the algorithms, each algorithm was trained and tested ten times. The performance of binary and multi-classification algorithms was evaluated using accuracy, while the effectiveness of the quantitative regression was assessed using Mean Absolute Error (MAE). The calculation methods for these metrics are as follows:

2 $$AC{C}_{binary}=\frac{TP+TN}{TP+TN+FP+FN}$$

3 $$AC{C}_{multi}=\frac{{\sum }_{i=1}^{12}{TM}_{i}}{{\sum }_{i=1}^{12}(T{M}_{i}+F{M}_{i})}$$

4 $$MAE=\frac{1}{n}\mathop{\sum }\limits_{i=1}^{n}| {y}_{rel}-{y}_{pre}| $$

where *TP* and *TN* represent correctly identified coal and coal-measure rock, respectively, while *FP* and *FN* denote incorrectly identified coal and coal-measure rock. Similarly, TM and FM denote the identification of correct and incorrect coal types. The variable *n* refers to the total volume of data recognized, *y*_*rel*_ represents the real Aad of the sample, and y_pre_ indicates the predicted Aad of the sample.

**Fig. 4:**
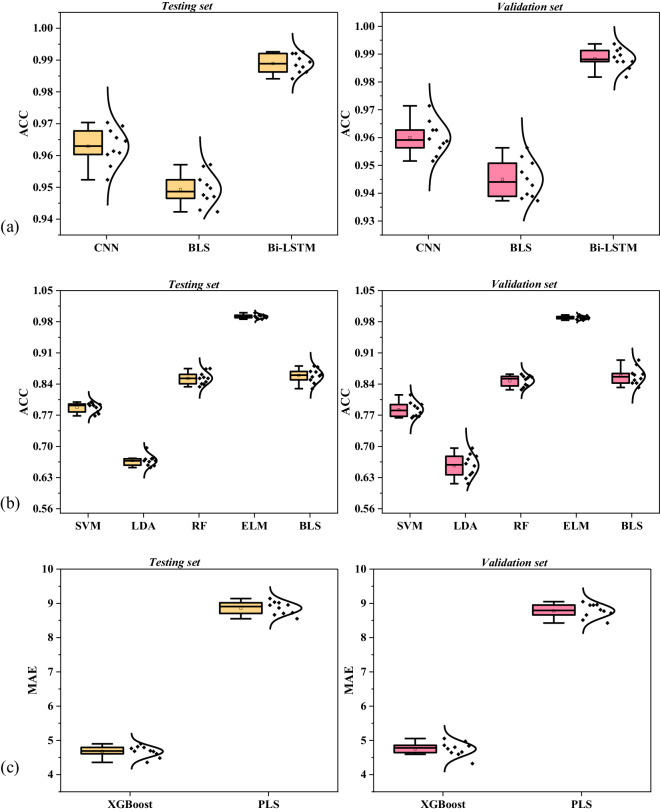
Accuracy and error for binary classification (**a**), multi-classification (**b**), and quantitative regression (**c**) tasks in testing set and validation set.

Fig. 4 displays the accuracy and error for binary classification, multi-classification, and quantitative regression tasks. In Fig. 4(a), the identification accuracy of all algorithms is no less than 94%, with the Bi-LSTM algorithm achieving the highest accuracy, because bi-LSTM can consider the correlation between wide-range wavelengths. It performed well in both the test and validation sets, reaching an average accuracy of 98.84%. In Fig. 4(b), due to the limitations of its linear classifier, the LDA algorithm performed poorly, whereas the ELM algorithm showed the highest and most stable accuracy, with an average accuracy of 98.89% in the validation set. The other algorithms also maintained a stable accuracy around 84%. In Fig. 4(c), XGBoost, with its ability to capture nonlinear relationships, achieved a lower error than to PLS, with an average MAE of 4.75% in the validation set.

In addition, we will further expand the coal and coal-measure rock near-infrared spectral dataset by adding a reflectance spectrum under the interference of external factors, such as dust, water mist, etc. This work will make the dataset universally applicable in various stages of coal mining. We also encourage other researchers in the coal mining field to expand and improve this dataset. The coal and coal-measure rock near-infrared reflectance spectrum collected in different conditions have significant implications for applying identification algorithms in practical work. The aim is to support further research and advancements in the intelligentization of coal mining.

## Data Availability

In this article, spectral acquisition, calibration, and data export were all completed using Avantes’ Avasoft software, with the software version being 7.8. The algorithms used for data validation were implemented using Python code, and the distribution version of the code can be obtained at the following website: https://github.com/smartLybo/Spectral-data-processing.git.
